# ABO-incompatible orthotopic heart transplant: a case report★

**DOI:** 10.1051/ject/2023009

**Published:** 2023-06-28

**Authors:** Ashlyn Strickland, Dafne Andrea Chianella, Minoo Kavarana, Andrew Savage

**Affiliations:** Medical University of South Carolina 171 Ashley Ave Charleston 29425 USA

**Keywords:** ABO-incompatible (ABOi), Heart transplantation, Pediatric, Congenital heart disease, Cardiopulmonary bypass, Exchange transfusion, Antibodies

## Abstract

*Background*: ABOi heart transplant has become routine for the majority of children <2 years old. An 8-month-old child with complex congenital heart disease presented to the Medical University of South Carolina Shawn Jenkins Children’s Hospital in need of transplantation. *Methods*: This case report describes the use of ABOi transplantation and describes the details of the total exchange transfusion prior to cardiopulmonary bypass. *Results*: After a successful intraoperative total exchange transfusion following the ABOi protocol, the patient’s isohemagglutinin titers were 1 VC on postoperative day (POD) 1, and isohemagglutinin titer was <1 VC on POD 14. The patient had no signs of rejection and continued to recover. *Conclusions*: Successful ABOi transplantation requires planning, an interdisciplinary approach, and clear closed-loop communication. Planning with the surgical and anesthesia teams is necessary for the hemodynamic stability of the patient during the total volume exchange as well as precautions put in place to ensure the blood products used in this procedure are correct. Planning with the lab and blood bank is also necessary to ensure they are prepared with enough blood products and can run isohemagglutinin titers.

## Background

ABO-incompatible (ABOi) heart transplantation was first performed in the late nineties by Toronto’s Hospital for Sick Children [1]. ABO isoagglutinin is a naturally occurring antibody that starts forming within 6 months of age and remains low through the first or second year of life. ABO antigen is expressed on red blood cells. A high isoagglutinin titer can cause an antibody-mediated rejection following transplantation. ABOi heart transplantation is a strategy performed on infants who have not yet developed relevant quantities of antibodies against donor blood groups (BG) [1] (Table 1). This transplant strategy reduced local waitlist mortality from > 50% to as low as 7% following routine acceptance of ABOi organs [2]. This is particularly advantageous for those patients in blood group O who have a small donor pool and are competing with all other blood group recipients for type O donor organs. Post-transplant survival is similar despite ABOi-listed children still showing a higher risk profile [3].

**Table 1:** ABO matching.

For recipient	Compatible organ donor blood groups	Incompatible
O	O	A, B, AB
A	A, O	B, AB
B	B, O	A, AB
AB	AB, B, A, O	–
For organ donor	Compatible recipient blood groups	Incompatible
O	O, A, B, AB	–
A	A, AB	O, B
B	B, AB	O, A
AB	AB	O, A, B

## Patient information

**Figure 1:**
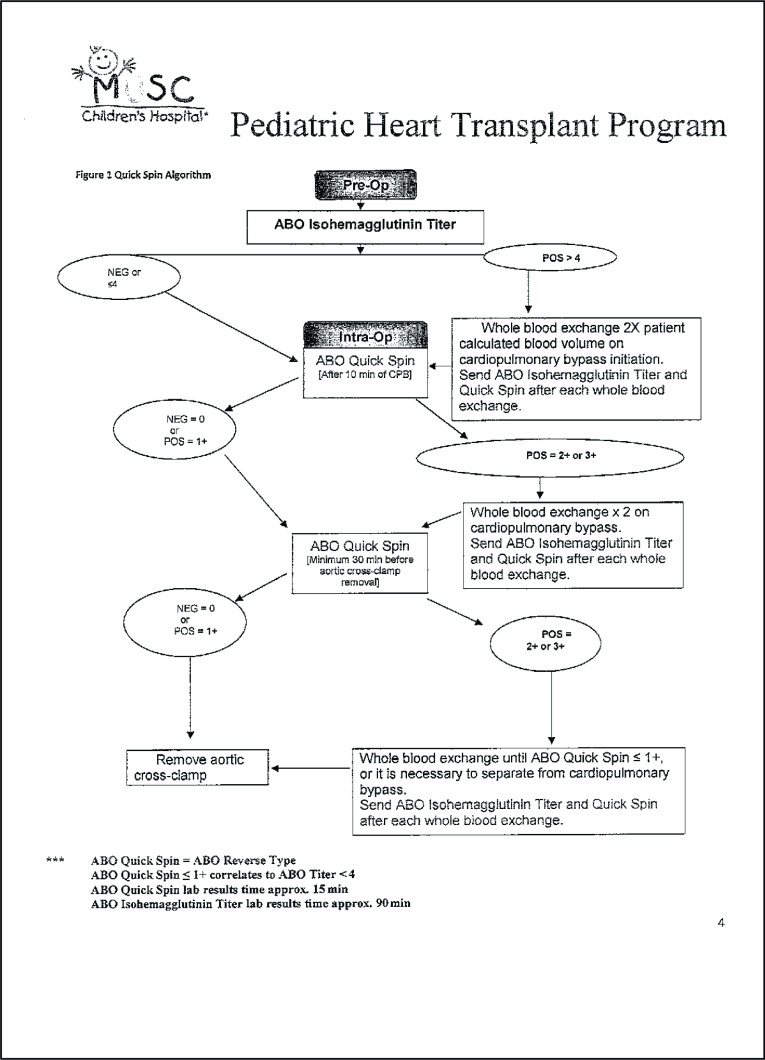
MUSC Children’s Hospital Pediatric Heart Transplant Program Quick Spin Algorithm guidelines for ABOi transplantation.

An 8-month-old female patient presented with NYHA Class IV congestive heart failure due to right ventricle dependent coronary circulation (RVDCC) and pulmonary atresia with an intact interventricular septum, and was indicated for an ABOi orthotopic heart transplant in early March of 2021 at the Medical University of South Carolina (MUSC). In early December 2020, she received a Berlin Heart^©^ (Berlin Heart GmbH, Berlin, Germany) left ventricular assist device (LVAD), and remained an inpatient. After several pump exchanges, she required a 25 mL pump averaging 2.0 L/min flow. Once the team confirmed an acceptable match, the bivalirudin was discontinued. The patient had no known allergies to the A^+^ blood type, the donor blood type was AB^+^. Preoperative lab work showed: Na 142 mEq/L, K 3.4 mEq/L, Cl 105 mmol/L, BUN 15 mg/dL, and creatinine 0.2 mg/dL. Hematocrit was 41%, hemoglobin was 14.3 g/dL, and the platelet count was 182 K/cumm. The patient was 6.86 kg and 68 cm with a BSA of 0.36 m2 calculated with the Haycock BSA Equation. At a 2.6 cardiac index, the full flow was estimated to be 947 mL/min for this patient. At 85 mL/kg the patient’s blood volume was calculated to be 583 mL. With a pump prime of 300 mL, total blood volume was added up to 883 mL. Due to a preoperative isohemagglutinin titer of 4 VC a whole blood two-time volume exchange transfusion was required equaling 1,766 mL. The decision to do a whole blood two-time volume exchange was made following The MUSC Children’s Hospital Pediatric Heart Transplant Program Quick Spin Algorithm guidelines for ABOi transplantation (Figure 1).

## Operative plan

The cardiopulmonary bypass (CPB) circuit was primed per MUSC ABOi pediatric perfusion guidelines with Plasmalyte A (Baxter Healthcare Corporation, Deerfield, IL, USA), 20 mEq of sodium bicarbonate (Hospira, Lake Forest, IL, USA), 428 mL of fresh frozen plasma (FFP) (2 units), 2,000 IU of heparin (Fresenius Kabi, Lake Zurich, IL, USA), 760 mL of washed A^+^ irradiated packed red blood cells (pRBCs) [2 units washed in a Fresenius Kabi CATS Plus^©^ (Terumo, Ann Arbor, MI, USA) cell saver on emergency wash setting], and 200 mg of CaCl (American Regent Inc. Shirley, NY, USA). The circuit was primed with A^+^ pRBC) to be ABO compatible with the recipient. All plasma components added to the pump must have no anti-A or anti-B antibodies to both donor and recipient. The cardiopulmonary bypass equipment used in this procedure consisted of a Sorin S5^©^ (LivaNova, London, UK) pump with Terumo^©^ Capiox cardiotomy reservoir and Capiox^©^ RX05 oxygenator (Terumo, Ann Arbor, MI, USA) and D736 arterial line filter (Terumo, Ann Arbor, MI, USA). Arterial and venous lines were ¼″ as appropriate for this patient’s full flow and volume status. The venous line was attached to a y connector leading to two separate Fresenius Kabi CATS Plus^©^ cell saver reservoirs put in place to hold the patient’s removed volume. Flow probes were used on the arterial line distal to the arterial line filter, as well as on the venous line leading to the removed volume reservoirs. The flow probes ensured an isovolumetric volume exchange. A 10 Fr DLP^©^ arterial cannula (Medtronic, Dublin, Ireland) was placed in the aorta, and a 16 Fr DLP^©^ venous cannula (Medtronic, Dublin, Ireland) was placed in the right atrium for the volume exchange, with a second 12 Fr DLP^©^ venous cannula (Medtronic, Dublin, Ireland) placed in the inferior vena cava (IVC) post volume exchange. Additional equipment used consisted of a CATS Fresenius^©^ cell saver, a Sorin Vanguard cardioplegia heat exchanger (LivaNova, London, UK), and Sorin^©^ 3T heater-cooler (LivaNova, London, UK).

A preoperative isohemagglutinin titer was measured with a result of 4 VC. Following The MUSC Children’s Hospital Pediatric Heart Transplant Program Quick Spin Algorithm guidelines for ABOi transplantation (Figure 1), an intraoperative whole blood two-time volume exchange was necessary. After sternotomy and adequate dissection and visualization, the MUSC protocol of 500 IU/kg of heparin loading dose was administered via anesthesia. Once an ACT >480 seconds was achieved, the aorta was cannulated. CPB suckers remained off until post volume exchange, and all volume sent to the cell saver would be discarded and the cell saver circuit replaced post exchange. The second cell saver was set up prior to the case, and on standby until after the exchange was performed. Once arterial cannulation was complete, the Berlin Heart was discontinued, and both inflow and outflow lines were clamped. The right atrium was cannulated and the volume exchange was initiated. A clamp was placed past the y connection on the venous line directing all returns into the exchange reservoirs. The pump reservoir volume was slowly infused via the roller head up the arterial line into the patient while gravity drainage and a clamp-controlled method were employed to initiate an isovolumetric exchange and maintain hemodynamic stability with pharmaceutical help from anesthesia to maintain pressures. Once approximately 1,700 mL were exchanged over 6 min, the total volume exchange was complete and the exchange reservoirs were clamped. Cardiopulmonary bypass was initiated, and suckers were turned on. All of the exchanged volumes were discarded and the cell saver was replaced with new sterile disposables.

Once on CPB, the patient was cooled to 32 °C and a 12 Fr DLP^©^ venous cannula (Medtronic, Dublin, Ireland) added in a 12 Fr DLP^©^ IVC drainage cannula was placed. Ten minutes post-CPB initiation, a second isohemagglutinin titer was sent to the lab and came back <1 VC indicating the lack of antibodies in the blood that will reperfuse the organ upon removal of the cross clamp, thus decreasing the potential for rejection. No further volume exchange was necessary at that time. Upon placing the aortic cross-clamp, 212 mL of antegrade Del Nido cardioplegia was administered to the recipient’s heart in the 1:4 blood: crystalloid ratio. Simultaneously, the donor’s heart was prepared and the recipient’s heart was removed. At 30 min prior to clamp removal, a third isohemagglutinin titer was sent to the lab that came back again as <1 VC, indicating the cross-clamp could be removed. Steroids were administered, 15 mg/kg methylprednisolone (Pfizer, New York, NY, USA), and the cross-clamp was removed, with a total donor cross-clamp time of 300 min and a recipient cross-clamp-time of 95 min. During rewarming 1,715 mg of 25% mannitol (Hospira Inc, Lake Forest, IL, USA) was given along with 200 mg CaCl (American Regent Inc. Shirley, NY, USA). After 204 min of bypass, the patient was successfully weaned from CPB, and atrial-venous modified ultrafiltration was performed for 10 min removing 300 mL of excess volume.

## Postoperative

The patient returned to the ICU on 5 mcg/kg/min of dopamine (Hospira, Lake Forest, USA), 0.03 mcg/kg/min of epinephrine (Par Pharmaceutical, Chestnut Ridge, USA), 0.5 mcg/kg/min of milrinone (West-Ward Pharmaceutical Corp., Eatontown, USA), and 0.5 mcg/kg/h of dexmedetomidine (Accord Healthcare, Inc, Durham, USA). On postoperative day 1, her isohemagglutinin titers were 1 VC. The patient continued on IV steroids (methylprednisolone) and IV mycophenolate mofetil (Genentech, San Francisco, USA) for immunosuppression. The patient continued to improve and two weeks postoperatively, the isohemagglutinin titer was <1 VC and there were no signs of rejection and continued to recover and was weaned off of sedation. Post-op day 23, the patient was extubated, eating, and comfortable, with cannulation sites healing well.

## Conclusion

Successful ABOi transplantation requires planning, an interdisciplinary approach, and clear closed-loop communications. Contact with the lab is necessary for timely intraoperative titer testing. The time-sensitive nature of the total exchange transfusions is critical, and cooperation from the operative lab technologist ensures accurate results. Planning with the surgical and anesthesia teams is necessary for the hemodynamic stability of the patient during the total volume exchange as well as precautions put in place to ensure the blood products used in this procedure are correct.

## Data Availability

All available data are incorporated into the article.
